# Cancer risk in microscopic colitis: a retrospective cohort study

**DOI:** 10.1186/s12876-018-0926-4

**Published:** 2019-01-05

**Authors:** Alexander Levy, Nienke Z. Borren, Benjamin Maxner, William Tan, Danielle Bellavance, Kyle Staller, Daniel Chung, Hamed Khalili, Ashwin N. Ananthakrishnan

**Affiliations:** 1 Division of Gastroenterology, Tufts Medical Center, Boston, MA 02111 USA; 2 Division of Gastroenterology, Massachusetts General Hospital, Boston, MA 02114 USA; 3 Harvard Medical School, Boston, MA 02115 USA

**Keywords:** Microscopic colitis, Cancer, Colorectal neoplasia, Adenoma, Lymphocytic colitis

## Abstract

**Background:**

The long-term natural history of microscopic colitis (MC) (collagenous colitis (CC), lymphocytic colitis (LC)), traditionally considered relapsing but non-progressive diseases, is poorly defined. Whether persistent histologic inflammation in such diseases is associated with an increased risk of colorectal neoplasia (CRN) or extracolonic cancers has not been robustly established.

**Methods:**

This retrospective cohort included diagnosed with MC at a referral center. Rates of CRN and extracolonic cancer were compared to patients undergoing screening colonoscopy (*n* = 306) and to the United States population using data from the Surveillance, Epidemiology, and End-Results (SEER) program. Standardized incidence ratios (SIR) and 95% confidence intervals were calculated and multivariable regression models used to identify the effect of MC diagnosis and severity on cancer risk.

**Results:**

Our study included 221 patients with microscopic colitis (112 CC, 109 LC) among whom 77% were women. Compared to the colonoscopy control population, MC was associated with similar odds of tubular adenoma (Odds ratio (OR) 1.07, 95% CI 0.69–1.66) or villous adenoma (OR 1.26, 95% CI 0.17–9.42). Compared to patients with a single episode of MC, those with 2 or more episodes had similar risk of colon cancer (OR 0.83, 95% CI 0.20–3.39) or tubular adenoma (OR 1.49 95% CI 0.83–2.67). We also identified no statistical increase in the rates of cancer in the MC population compared to US-SEER data.

**Conclusion:**

Microscopic colitis was not associated with increased risk of CRN and extracolonic cancers when compared to controls undergoing colonoscopy or the US SEER population.

## Background

Microscopic colitis (MC) is a chronic, inflammatory colitis that commonly presents as watery diarrhea and is a source of morbidity, particularly in older individuals. The incidence of MC has been reported to be between 1 and 24 per 100,000 person-years in North America and Europe from population-based studies [1–6]. It comprises two subtypes, collagenous colitis (CC) and lymphocytic colitis (LC) that share many clinical and epidemiological characteristics including female predominance and a normal colonoscopic mucosal appearance. However, they are distinguished by their characteristic histologic features; an increase in intra-epithelial lymphocytosis (> 20/100 epithelial cells) in LC and a thickened sub-epithelial collagen band (> 10 μm) in CC [7, 8]. Both conditions may be associated with a mixed infiltrate of acute and chronic inflammatory cells in the lamina propria [8].

Traditionally considered relapsing but non-progressive diseases, the long-term natural history of MC is not well defined. Simple anti-diarrheal therapy with anti-motility agents are considered equally a first line for treatment of MC as inflammation directed therapies such as budesonide and systemic steroids [9]. As a recognition of its non-progressive nature, resolution of histologic inflammation is not necessary or aimed for as a therapeutic target [10]. It is well established that for various chronic inflammatory diseases involving the gastrointestinal and hepatobiliary tracts including inflammatory bowel diseases (IBD; Crohn’s disease (CD), ulcerative colitis (UC), celiac disease, or primary sclerosing cholangitis, there is an increase in risk of cancer in the target organ that may be independently associated with persistent histologic activity [11–16]. In addition, such inflammatory diseases have also been associated with an increased risk of various extra-intestinal malignancies, the mechanisms of which have not been robustly defined [14, 17, 18]. Whether persistent chronic inflammation in microscopic colitis is associated with an increased risk of colorectal neoplasms (CRN) including cancer (CRC) has not been well established. The few studies that have examined this have been limited by small cohort size, short duration of follow-up, and lack of adjustment for severity of MC [19, 20]. Additionally, whether there is an increase in risk of extraintestinal cancers in MC has not been previously established.

The aims of our study were as follows: (1) to examine the life-time risk of colorectal cancer (CRC) in patients with MC when compared to the general population in the United States (US) or to similar patients undergoing colonoscopic screening; and (2) to define if there is an increase in life-time risk of extracolonic cancers in patients with MC.

## Methods

### Study population

We performed a retrospective cohort study of patients receiving care for microscopic colitis at a tertiary referral center. Patients with a confirmed diagnosis of microscopic colitis as determined by clinical, endoscopic, and histologic criteria were eligible for inclusion in our study. First, the Partners Research Patient Data Repository (RPDR) was queried for all possible patients with a diagnosis of MC based on the presence of one or more International Classification of Diseases (ICD) (9th edition) (ICD-9) codes for other or unspecified colitis in combination with at least one colonoscopic evaluation within our system. Free text search was performed among all the pathology reports to identify those with mentions of “microscopic colitis”, “lymphocytic colitis”, or “collagenous colitis”. Manual chart review was performed by one of the study investigators (AL) for all such patients, and those where the diagnosis of MC, LC, or CC could be confirmed were included in our study.

### Study outcomes and covariates

Our primary study outcome was the development of colorectal cancer and colonic adenomas. Secondary outcomes were the development of extra-intestinal cancers. Manual review of the charts was performed to identified each of these study outcomes. After confirming diagnosis patient demographics, including age, sex and smoking history were noted. Disease characteristics, including MC subtype, treatment history, remission, and recurrence data were recorded.

### Control populations

To determine if the risk of cancer was increased in patients with MC, we used two control populations. First, we compared the observed rates of cancer in our MC cohort to data from the Surveillance, Epidemiology, and End-Results (SEER database). For each cancer, we determined the expected number of cases in our population by applying the cumulative age- and gender-specific incidence rates at 10-year intervals. SEER is a population-based cancer registry in the United States that collects incidence and prevalence information for every cancer and covers an estimated 28% of the US population.

The second control population was the GI Disease and Endoscopy Registry (GIDER) at Massachusetts General Hospital. In brief, this is a prospective registry of patients undergoing colonoscopic screening within the GI practices at MGH. Patients with prior colon cancer or known gastrointestinal disease are excluded from enrollment in the cohort. Upon providing informed consent, patients provided detailed information on health history including demographics and lifestyle information, medical co-morbidities, and history of extra-colonic malignancies.

### Statistical analysis

Continuous variables were summarized using means and standard deviations (or median with interquartile ranges when skewed) while categorical variables were expressed as proportions. T-tests and chi-square tests were used to compare continuous and categorical variables respectively. Multivariable logistic regression was performed to identify if characteristics of MC in our study population were independent predictive of risk of colonic neoplasia and extra-colonic malignancies. A two-sided *p*-value < 0.05 indicated independent statistical significance in such analysis. Standardized incidence ratios and corresponding 95% confidence intervals were calculated to examine if there is an excess risk of malignancies in our MC cohort compared to the population from the SEER database. Multivariable regression models were used to examine if a diagnosis of microscopic colitis was associated with increased risk of colon and extra-colonic neoplasia compared to controls from the MGH GIDER screening cohort. The study was approved by the Institutional Review Board of Partners Healthcare. All statistical analysis was performed using Stata 13.1 (StataCorp, College Station, TX).

## Results

### Study cohort

Our study cohort included 221 patients with MC among whom 116 had a diagnosis of lymphocytic colitis (53%) while the remaining 105 patients had collagenous colitis (48%). The median age at diagnosis was 64 years (interquartile range (IQR) 54–71 years) and over three-quarters of the cohort were women (*n* = 171, 77%). Approximately 50% of the cohort had used budesonide, and 7 and 5% respectively had used immunomodulator and anti-TNF biologic therapy. The mean duration of follow-up with microscopic colitis in our center was 3.5 years (range 1–19 years); however as several patients had been diagnosed prior to establishing care with us, this does not represent true duration of disease. Thirty patients (14%) with microscopic colitis had a family history of colorectal neoplasia. Table 1 compares the characteristics of those with LC and CC. There was no difference in age, gender, or racial/ethnic distribution between the two groups. Those with collagenous colitis were more likely to have used non-steroidal anti-inflammatory drugs (23% vs. 12%) but were similar in their use of aspirin or COX-2 inhibitors. Patients with microscopic colitis were more likely to be using aspirin (36% vs 8%) but less likely to be current users of NSAIDs (27% vs 17%) (*p* <  0.05 for both) when compared to controls. A larger proportion of patients with CC used budesonide or prednisone when compared to those with LC but this difference was not statistically significant. Immunomodulator or biologic use was infrequent in both groups (9% in LC, 13% in CC, *p* = 0.26). Patients with CC had a trend towards a greater number of mean episodes of relapse (1.8 vs. 1.4, *p* = 0.06).

**Table 1:** Comparative characteristics of patients with lymphocytic colitis and collagenous colitis

Characteristic	Lymphocytic colitis	Microscopic colitis	*p*-value
Mean age at diagnosis (in years) (SD)	59.5 (18.1)	62.5 (13.1)	0.15
Gender			0.13
Female	73%	82%	
Male	27%	18%	
Race / Ethnicity			0.50
White	93%	95%	
Non-white	7%	5%	
Ever smoking	47%	43%	0.58
Aspirin use	37%	33%	0.56
NSAID use	12%	23%	0.03
Treatment for MC			
Budesonide	45%	55%	0.12
Prednisone	7%	13%	0.11
Cholestyramine	12%	19%	0.15
Immunosuppressant	9%	13%	0.26
Mean number of episodes (SD)	1.77 (1.68)	1.40 (1.25)	0.059

### Distribution of colorectal neoplasia and comparison to controls

**Fig. 1:**
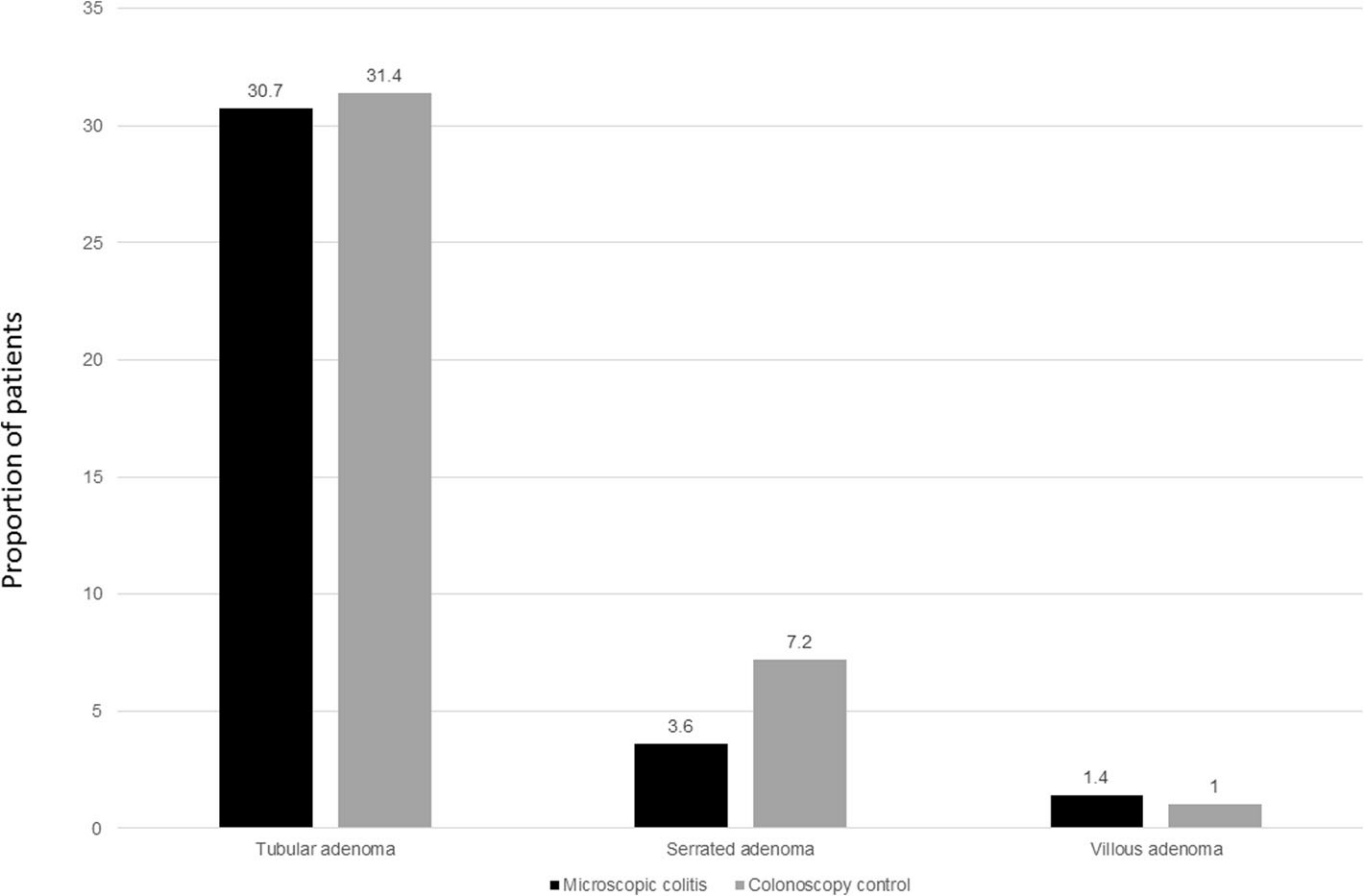
Frequency of colorectal polyps in microscopic colitis (*n* = 221) compared to controls undergoing colonoscopy (*n* = 306)

Table 2 compares the characteristics of patients with microscopic colitis compared to colonoscopy controls. Among the MC cohort, sixty-eight (31%) patients had a tubular adenoma, 8 had a serrated adenoma (4%), 3 had villous adenomas (1%) and nine developed colon cancer (4%). These rates were similar to that observed in the colonoscopic screening control population where the rates of tubular adenomas, serrated adenomas, and villous adenomas were 31.4, 7.2 and 1% respectively (*p* = NS for all comparisons) (Fig. 1). On multivariable analysis, compared to the general colonoscopy control population, adjusting for age, gender, smoking, and body mass index, microscopic colitis was associated with similar odds of tubular adenoma (Odds ratio (OR) 1.07, 95% CI 0.69–1.66) or villous adenoma (OR 1.26, 95% CI 0.17–9.42). There was no statistically significant difference in frequency of neoplasia between lymphocytic and collagenous colitis.

**Table 2:** Comparison of characteristics of patients with microscopic colitis and controls undergoing screening colonoscopy

Characteristic	Microscopic colitis (*n* = 221)	Controls (*n* = 306)	*p*-value
Mean age (in years) (SD)	65.7 (15.4)	61.0 (9.9)	< 0.001
Gender			< 0.001
Female	77%	50%	
Male	23%	50%	
Mean Body mass index (in kg/m2) (SD)	26.0 (5.2)	29.0 (5.9)	< 0.001
Ever smoking	45%	31%	0.001

A total of 77 patients (35%) in our MC cohort had at least one colorectal neoplasia or cancer. On multivariable analysis, the only independent risk factor for development of CRN was older age. Each 1 year increase in age was associated with a 3% increase in odds of CRN (OR 1.03, 95% CI 1.01–1.06). Severity of MC quantified by number of discrete episodes was not predictive of CRN. Compared to patients with a single episode of MC, those with 2 or more episodes had similar risk of colon cancer (OR 0.83, 95% CI 0.20–3.39) or tubular adenoma (OR 1.49 95% CI 0.83–2.67).

### Comparison to the SEER population

Table 3 presents the SIRs for colonic and extra-colonic cancers in our MC cohort when compared to the SEER population. Compared to the general US population, MC was not associated with an increased risk of CRC in either men (SIR 1.59, 95% CI 0.27–5.24) or women (SIR 1.97, 95% CI 0.86–3.89). Similarly, there was no increase in other gastrointestinal cancers, lung, breast, or thyroid cancer in patients with MC compared to the general US population. There was a numerically higher but statistically insignificant risk of thyroid cancer in women with MC (SIR 2.20, 95% CI 0.89–4.57).

**Table 3:** Standardized incidence ratios and confidence interval for colonic and extracolonic cancers in microscopic colitis compared to US SEER population

	Standardized incidence ratio (95% CI)	
Cancer type	Women	Men
Colorectal cancer	1.97 (0.86–3.89)	1.59 (0.27–5.24)
Gastric cancer	0 (0–5.58)	3.75 (0.19–18.51)
Pancreatic cancer	1.81 (0.30–5.98)	2.55 (0.13–12.59)
Liver cancer	0 (0–6.25)	0 (0–7.61)
Lung cancer	1.26 (0.51–2.62)	0.60 (0.03–2.95)
Breast cancer	1.30 (0.83–1.93)	–
Thyroid cancer	2.20 (0.89–4.57)	0 (0–12.82)
Bone cancer	0 (0–30.02)	0 (0–87.09)
Melanoma	1.76 (0.77–3.47)	0.68 (0.03–3.37)

*CI* confidence interval, *SEER* Surveillance, Epidemiology, and end-result

## Discussion

Microscopic colitis is an important source of morbidity and a frequent cause of watery diarrhea in the elderly. Characterized by relapses and periods of remission, there is limited data on the long-term outcome of microscopic colitis, particularly in relation to risk of malignancies given the persistent histologic inflammation and symptom-based approach to therapy. Here, using a large retrospective cohort of patients with microscopic colitis with long-term follow-up, we observe, reassuringly, that there is no increase in risk of colonic or extra-colonic malignancy in these patients.

Chronic inflammation in the gastrointestinal tract has been associated with increased risk of malignancy in several diseases, most notably increased risk of CRC with ulcerative colitis and Crohn’s disease [12, 14, 21]. In addition, small bowel CD is associated with a higher risk of small bowel adenocarcinoma, refractory celiac disease characterized by persistent lymphocytosis and villous blunting with enteropathic T-cell lymphoma, and primary sclerosing cholangitis with cholangiocarcinoma and gall bladder cancer [16, 17, 22, 23]. Common among these diseases is a persistent inflammation in the affected tissue. Indeed, independent of other risk factors, the persistence of histologic inflammation has been hypothesized to contribute to an increased risk of dysplasia and CRC in patients with IBD [15, 24].

Evidence also supports that persistent inflammatory diseases are associated with an increase in risk of systemic malignancies. Multiple studies have noted an increase in risk of various cancers including Non-Hodgkin’s lymphoma, urinary tract cancer, female breast cancer, prostate, and lung cancer in patients with IBD compared to controls [25–29]. A study of over 30,000 Finnish patients with celiac disease identified increased risk of basal cell skin cancer, non-Hodgkin’s lymphoma, small intestinal, and colon cancer compared to controls [30]; Swedish population-based registry data also suggested an increase in hepatobiliary and pancreatic cancers in such patients [31].

In the context of this data, it is notable and reassuring that microscopic colitis which is treated primarily symptomatically and where resolution of histologic inflammation is not a therapeutic target was not associated with a higher risk of colonic or extra-colonic malignancies. Only a few previous studies have examined cancer risk in patients with MC. A multi-center study of patients with chronic diarrhea undergoing colonoscopy showed an inverse association between microscopic colitis and neoplastic colon polyps; however, this study was limited by a cross-sectional analysis and small number of included participants [32]. Yen et al. examined the rates of CRC in 647 patients with MC and noted a reduced risk of CRC when compared to controls [20]. An analysis of 117 patients with collagenous colitis from Johns Hopkins found no increase in risk of CRC but noted an increased relative risk of lung cancer [19]. There may be a few possible explanations for why MC may not be associated with risk of cancer, particularly CRC. It is possible that the older age of onset (and consequently shorter disease duration), gender (predominantly female), and ethnicity (primarily Caucasian population) may account for lower CRC risk in MC patients, as the highest risk of CRC is often noted to be in non-Hispanic black men [26]. However, our gender-stratified analysis did not demonstrate a risk in either gender. Whereas sporadic colorectal cancer is known to follow an adenoma-carcinoma sequence, the result of chromosomal and microsatellite instability, colitis-associated carcinoma is thought to occur through a progression of dysplasia to carcinoma, with analogous genetic mutations occurring at different times and frequency along the carcinoma pathway [24]. Oxidative stress coupled with chronic inflammation may contribute to neoplastic transformation via DNA damage and subsequent activation of pro-oncogenic genes and inhibition of tumor suppressor genes [15, 25]. While merely conjecture, one may hypothesize that the severity or mechanism of inflammation in MC does not initiate a similar dysplasia-carcinoma pathway as seen in IBD. Treatment practices may also help explain the differences in cancer risk between IBD and MC. Prolonged immunosuppression, a cornerstone of IBD treatment and associated with certain cancers is not commonly employed in MC.

Our study has several strengths. Few previous studies have examined the long-term risk of MC and both CRC and extracolonic cancers. To our knowledge, there has also not been prior examination of whether severity of MC modified such as risk. As controls, we used both a screening colonoscopy population as well as data from SEER which reduces institutional bias. Both cases of MC and cancer outcomes were confirmed by medical record review by study investigators.

We readily acknowledge several limitations to our study. The follow up period for cancer included a span both preceding and following the diagnosis of microscopic colitis We adopted this approach rather than relying on time since diagnosis of MC for various reasons. First, microscopic colitis is often insidious and many patients have many years of diarrhea before undergoing the diagnostic colonoscopy. Thus, true onset of disease is difficult to establish. Second, some patients had their diagnosis of microscopic colitis prior to establishing care with us and consequently duration of disease was unavailable. Third, cancer history in both our screening control population or the SEER data estimates lifetime cumulative risk rather than over person-time of follow-up. To ensure comparability between all cohorts, we decided to examine life-time cancer risk as our primary outcome. However, because of these limitations, a true causal association cannot be demonstrated. Nevertheless, a null association is reassuring for an absence of a significantly elevated risk. Being based at a referral center, our cohort of MC may not be representative of the severity of disease noted in the general population and make be skewed towards more severe disease. However, fewer than 10% of our cohort were on immunosuppressive therapy and there is no prior data that severity of MC influences cancer risk. While our database captures diagnoses rendered at any Partners healthcare facility, we may not have comprehensively captured cancer diagnosis and care that occurred entirely outside our health system. The sample size, while still representing one of the largest studies to examine risk of cancer in microscopic colitis, limited statistical power to examine differences, particularly in rare extracolonic cancers. We also did not have full information on duration of disease, persistence of histologic activity and intensity of colorectal neoplasia surveillance in our cohort of microscopic colitis, and there is no validated definition for severity of microscopic colitis. As neither duration of MC nor histologic activity has been previously associated with risk of colorectal or extracolonic neoplasia, we do not believe this to be a significant limitation.

## Conclusions

In conclusion, we demonstrate that microscopic colitis is not associated with increase in risk of colonic or extra-colonic cancers. This provides reassurance to both patients with MC as well as providers involved in their care. However, it also intriguingly provides a stimulus for further research into why the persistence of inflammation in MC is not associated with risk of cancer when compared to other chronic gastrointestinal inflammatory diseases.

## Abbreviations

CRC: Colorectal cancer
IBD: Inflammatory bowel diseases
MC: Microscopic colitis
SIR: Standardized incidence ratio
